# Influence of tacrolimus metabolism rate on BKV infection after kidney transplantation

**DOI:** 10.1038/srep32273

**Published:** 2016-08-30

**Authors:** Gerold Thölking, Christina Schmidt, Raphael Koch, Katharina Schuette-Nuetgen, Dirk Pabst, Heiner Wolters, Iyad Kabar, Anna Hüsing, Hermann Pavenstädt, Stefan Reuter, Barbara Suwelack

**Affiliations:** 1 Department of Medicine D, Division of General Internal Medicine, Nephrology and Rheumatology, University Hospital of Münster, Münster, Germany; 2 Institute of Biostatistics and Clinical Research, University of Münster, Münster, Germany; 3 Department of General Surgery, University Hospital of Münster, Münster, Germany; 4 Department of Transplant Medicine, University Hospital of Münster, Münster, Germany

## Abstract

Immunosuppression is the major risk factor for BK virus nephropathy (BKVN) after renal transplantation (RTx). As the individual tacrolimus (Tac) metabolism rate correlates with Tac side effects, we hypothesized that Tac metabolism might also influence the BKV infection risk. In this case-control study RTx patients with BK viremia within 4 years after RTx (BKV group) were compared with a BKV negative control group. The Tac metabolism rate expressed as the blood concentration normalized by the daily dose (C/D ratio) was applied to assess the Tac metabolism rate. BK viremia was detected in 86 patients after a median time of 6 (0–36) months after RTx. BKV positive patients showed lower Tac C/D ratios at 1, 3 and 6 months after RTx and were classified as fast Tac metabolizers. 8 of 86 patients with BK viremia had histologically proven BKN and a higher median maximum viral load than BKV patients without BKN (441,000 vs. 18,572 copies/mL). We conclude from our data that fast Tac metabolism (C/D ratio <1.05) is associated with BK viremia after RTx. Calculation of the Tac C/D ratio early after RTx, may assist transplant clinicians to identify patients at risk and to choose the optimal immunosuppressive regimen.

While rates of acute rejection (AR) have declined during the last decades due to more effective immunosuppressive regimens^1^, BK virus (BKV) infection has become an emerging problem after renal transplantation (RTx)^2^. BK viremia occurs in 10–15% 3,4,5 with an increasing incidence of BKV-associated nephropathy (BKN) in more than 6% of patients by 5 years after transplantation^2^. Graft loss due to BKN has been reported in 10–80% of cases^6^,^7^. If BKV infection is associated with higher rates of acute rejection is still a matter of debate^3^,^6^,^8^.

Several risk factors for BKV infection including patient’s characteristics, quality of the allograft and archetype strain of the virus have been discussed^6^,^9^. Especially, modifiable risk factors of BKN such as intensity or type of immunosuppression are of interest to transplant physicians^2^,^4^,^10^. Immunosuppressive regimens including tacrolimus (Tac) have been shown to be associated with higher rates of BKV infections^2^,^11^. The individual Tac metabolism rate can be estimated by blood concentration (C) normalized by the dose (D) and has been shown to be related to side effects of the drug. We recently showed that fast Tac metabolizers (low C/D ratio) had a reduced renal function 24 months after RTx and 36 months after LTx, respectively^12^,^13^. This finding might be explained by higher Tac peak levels early after drug intake leading to a higher drug exposure (area under the curve) for these patients. The aim of this study was to analyze whether fast Tac metabolism is also a risk factor for BKV infection.

## Patients and Methods

### Study enrollment and detection of BKV and BKN

In this case-control study, 707 patients that underwent RTx from January 2005 until December 2011 were screened for BK viremia. Screening was performed monthly during the first 6 months after RTx, every second month during months 6–12, and on indication. Peripheral EDTA-blood was collected from each patient to assess BKV DNA by nested polymerase chain reaction (GeneProof BK Virus (BKV) PCR Kit, GeneProof a.s., Brno, Czech Republic). Any positive BKV PCR was considered as BKV infection. Patients with a viral load of ≥7,000 copies/mL^3^ or a relevant rise in creatinine (≥0.3 mg/dL) were subjected to kidney biopsy to distinguish BKN from other causes of graft failure. Biopsies were evaluated by one pathologist and BKN was diagnosed if interstitial nephritis and positive staining for SV40 antigen were present.

Of 95 patients who developed BKV infection after RTx, 9 patients deceased during the four year follow-up period and were excluded from the analysis. 86 BKV negative patients that underwent RTx during the same time period were randomly chosen as controls.

Immunosuppression consisted of a calcineurin inhibitor (CNI) like Tac twice daily (Prograf), Tac extended release formulation (Advagraf) or cyclosporine A (Sandimmun) respectively, MMF (CellCept) and prednisolone (Soludecortin H). Induction therapy with basiliximab (Simulect) was administered at day 0 and 4 after RTx.

Recipient and donor data were taken from the patient’s files. Transplant specific characteristics as HLA MM (human leukocyte antigen mismatches), panel reactive antibodies (PRA), attendance in the European Senior Program (ESP), blood type, living donor transplantations, number of prior RTx, cold and warm ischemia times, cytomegalovirus (CMV) status before transplantation, CMV infections (considered relevant >1.000 copies/mL) were documented.

Prior to analysis, data of all patients were anonymized and de-identified. The local ethics committee (Ethik Kommission der Ärtzekammer Westfalen-Lippe und der Medizinischen Fakultät der Westfälischen Wilhelms-Universität, No. 2014-381-f-N) approved the study. Methods in this study were carried out in accordance with the current transplantation guidelines and the Declarations of Istanbul and Helsinki. Written informed consent was given by all participants at the time of transplantation for recording their clinical data.

### Tacrolimus metabolism rate

Tac metabolism rates were calculated at month 1, 3 and 6 after RTx by dividing the Tac blood trough concentration (C) by the corresponding daily Tac dose (D) as previously published^12^:
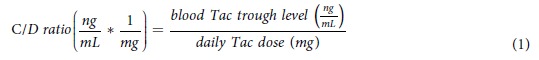


As defined previously, patients with a Tac C/D ratio <1.05 ng/mL*1/mg were characterized as fast metabolizers^12^. Patients with a C/D ratio of 1.05–1.54 ng/mL*1/mg or a C/D ratio ≥1.55 ng/mL*1/mg were defined as intermediate metabolizers and slow metabolizers, respectively. For reason of simplification, intermediate and slow metabolizers were summarized as slower metabolizers in this study.

### Clinical chemistry

Whole blood was analyzed for creatinine (enzymatic assay; Creatinine-Pap, Roche Diagnostics, Mannheim, Germany) and renal function was determined by eGFR calculation using the 4-variabel modification of diet in renal disease (MDRD) study at time of first BKV DNA detection and 12 months thereafter.

### Statistical analyses

Statistical analyses were performed using IBM SPSS^®^ Statistics 22 for Windows (IBM Corporation, Somers, NY, USA) and SAS software, version 9.4 of the SAS System for Windows (SAS Institute, Cary, NC, USA). Inferential statistics were intended to be exploratory, not confirmatory. P-values represent a metric measure of evidence against the respective null hypothesis and were used only to generate new hypotheses. Therefore, neither global nor local significance levels were determined, and no adjustment for multiplicity was applied. P-values ≤ 0.05 were considered as statistically noticeable.

Standard univariate statistical analyses were applied. Categorical variables are shown as absolute and relative frequencies. Fisher’s exact tests were used to quantify the evidence between categorical variables. Normal-distributed continuous variables are shown as mean ± standard deviation and not normal-distributed continuous variables as median [minimum – maximum]. Groups were compared using Student’s t-test for normally distributed data, Mann–Whitney U tests for non-normal data and Fisher’s exact tests for categorical variables. Multivariable logistic regression analysis was conducted for selected variables as age at RTx (years), CMV risk status and log-transformed Tac C/D ratio (1 month after RTx) to estimate the influence on the development of BKV infection within 4 years after RTx and to adjust for confounders. Results are presented as odds ratios (OR) and corresponding 95% confidence intervals (95% CI). Time until switch from initial Tac immunosuppression and corresponding event probabilities were estimated using Kaplan-Meier method.

## Results

### Study population

BKV DNA was detected in 95 RTx recipients. Due to the death of 9 BKV positive patients during the four year follow-up, 86 patients were included as cases in this study (BKV group) and compared to 86 BKV negative control patients. Although mean age was 3 years higher in the BKV group, univariate analysis did not show a noticeable difference (53.4 ± 13.2 vs. 50.4 ± 14.6 years; P = 0.172; Table 1). The logistic regression analysis also did not show an influence of patient’s age on BKV risk in this study (P = 0.067, Table 2). There was no difference in patients’ gender between both groups (P = 0.873).

In the BKV group more patients had a CMV high risk status (D+/R-) than in the control group (30.2% vs. 9.3%, P = 0.001, Fisher’s exact test, Table 1) and multivariable logistic regression analysis confirmed CMV high risk status to be a risk factor for BKV infection compared to a CMV intermediate risk status (P = 0.001, OR 6.03, 95% CI 2.17, 16.78, Table 2). Numbers of CMV infections and CMV diseases were similar in both groups (16.3% vs. 16.3% and 11.6% vs. 11.6% respectively). Half of CMV infections in the BKV group (7/14) had occurred before BKV detection (Table 1).

### Drug doses and blood levels

**Figure 1:**
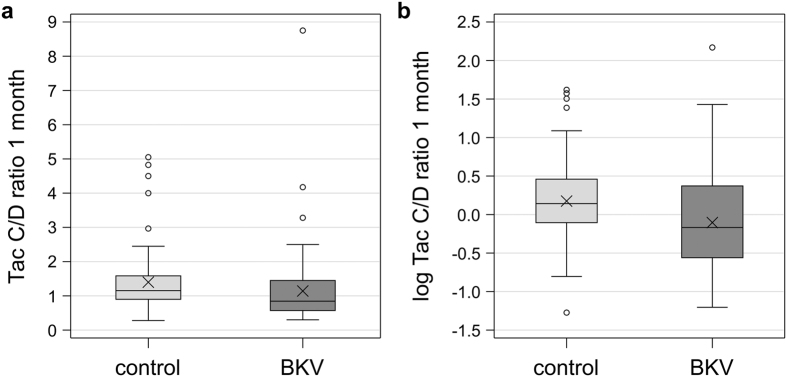
Boxplots of the Tac C/D ratio 1 month after renal transplantation (RTx). **(A)** The BKV group shows a noticeably lower C/D ratio compared to the control (0.85 (0.30–8.75) vs. 1.15 (0.28–5.05) ng/mL*1/mg; P = 0.002. **(B)** The Tac C/D ratio 1 month after RTx was log-transformed (natural logarithmic) to achieve equal intervals between C/D ratio units (−0.17 (−1.20–2.17) BVK group vs. 0.14 (−1.27–1.62) control group; P = 0.002). Symbol X marks the mean.

Table 3 shows patient’s drug doses and blood levels. Tac twice daily was initially administered in 75 of 86 (87.2%) patients in the BKV group and in 79 of 86 (91.9%) patients in the control group. Daily Tac doses were not different in both groups at 1, 3, and 6 months (P = 0.064; P = 0.135; P = 0.082, respectively) while Tac mean trough levels were about 1 ng/mL higher in the control group compared to the BKV group at 1, 3 and 6 months (P = 0.017; P = 0.149; P = 0.012, respectively). In the BKV group, the Tac C/D ratio was noticeably lower for all time points examined (P = 0.002; P = 0.017; P = 0.001, respectively). Figure 1 shows the distribution of the C/D ratio and the log-transformed values 1 month after RTx in both groups. Accordingly, multivariable logistic regression identified a low Tac C/D ratio (fast Tac metabolism) 1 month after RTx to be associated with BKV infection. Per unit increase of the log-transformed Tac C/D ratio, the odds decrease by OR 0.37 (95% CI 0.20, 0.71). Furthermore, the BKV group encountered noticeably more fast Tac metabolizers (C/D ratio <1.05 ng/mL*1/mg) at month 1 (Table 3; P = 0.002). Since prednisolone is known to influence Tac metabolism^14^, prednisolone doses were collected 1, 3 and 6 months after RTx. Median prednisolone doses were similar in both groups at all time points, although due to a greater range, a noticeable higher prednisolone dose was found 3 months after RTx in the BKV group (P = 0.018; Table 3).

### BK viremia and BKN

BKV infection was detected after a median time of 6 (0–36 months; Table 4). The median initial BK viral load was 5,540 (91–8,600,000) copies/mL and maximum viral load was 25,446 (91–196,000,000) copies/mL. Patients experiencing BKN showed higher initial and maximum viral loads (Table 4). MMF was stopped in nearly half of the patients (44/86) after BKV detection and was reduced in all other patients. Patients with BKN were predominantly fast metabolizers with a median Tac C/D ratio of 0.78 ng/mL*1/mg.

### Renal function

Renal function (eGFR) at the time of initial BKV DNA detection and 12 months thereafter did not differ between the two groups (36.9 ± 13.1 vs. 36.3 ± 13.4 mL/min/1.73 m^2^). Patients with BKN, however, showed a slightly reduced eGFR 12 months after initial BKV detection when compared to controls (34.1 ± 15.4 vs. 31.8 ± 15.5 mL/min/1.73 m^2^).

### Adverse events

**Figure 2:**
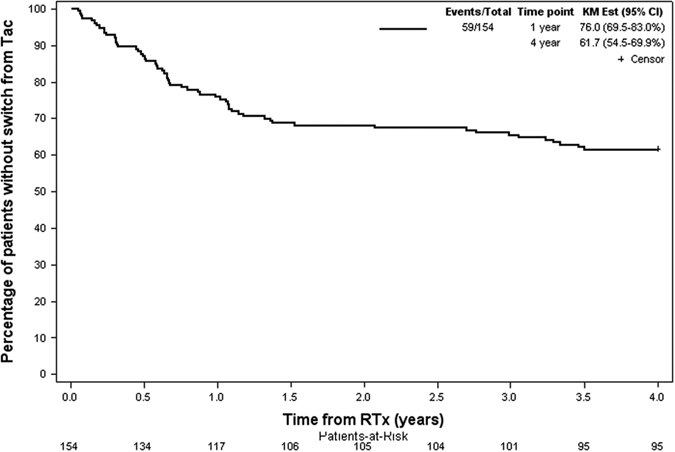
Kaplan-Meier plot of time until switch from Tac. Percentage of patients without switch from Tac are shown on the y-axis. All patients reached a 4 year follow-up and patients without switch were censored at four years. In the control group, 27.8% (22/79) of patients were switched from Tac to another immunosuppressive drug compared to 49% (37/75) in the BKV group. The main cause of switch from Tac during the first year after renal transplantation was BKV infection (Table 5). Abbreviations: KM Est, Kaplan-Meier estimator; RTx, renal transplantation.

Table 5 shows adverse events that occurred during the observational period. Rates of transplant loss were equal in both groups. Immunosuppression was switched from Tac twice daily to other regimens more often in the BKV group. The main cause for Tac withdrawal was BKV infection (Fig. 2).

## Discussion

In the present case-control study 86 patients who developed BKV infection within four years after RTx were compared to 86 BKV negative controls. BKV infection was associated with a fast Tac metabolism at 1 month after RTx. Furthermore, CMV high risk constellation (D+/R−) was identified as a risk factor for BKV infection.

BKV infection has become an important issue after RTx and several attempts to identify risk factors predisposing for posttransplant BKV infection have been made during the last years. Various parameters like immunosuppression, recipient characteristics, graft quality and virus specific features have been discussed to have an influence on the development of BKV infection.

The role of immunosuppression for the development of BK viremia is still not clear and data is limited. Dadhania *et al*. suggested steroid maintenance to be an independent risk factor for BKV replication^10^. Montero *et al*. summarized data of one randomized controlled trial and 13 cohort studies on pancreas and pancreas plus kidney transplant patients and concluded that steroid-sparing and withdrawal strategies reduced the risk of BKV infection^15^. In our cohort, both groups showed similar prednisolone doses 1 month after RTx. However, at 3 months after RTx, prednisolone doses were higher in the BKV group suggesting that higher prednisolone doses might be associated with an increased risk of BKV infection. This is in line with data by Hirsch *et al*. who reported that BK viremia 6 months after transplantation was independently associated with higher steroid exposure in the first three posttransplant months^11^.

Increased BKV infections in patients receiving a Tac-based immunosuppressive therapy were ascribed to the stronger immunosuppressive potency of Tac compared to other immunosuppressive drugs such as mammalian target of rapamycin inhibitor (mTORi) or cyclosporine A, or different BKV-promoting or –inhibiting features in the mode of action of the respective drug^11^. E.g. Tac was shown to directly stimulate BKV replication via FKBP-12 binding^16^. However, in our study, Tac mean daily doses did not differ between the groups and Tac mean trough levels were about 1 ng/mL higher in the control group compared to the BKV group.

Therefore, we hypothesized that a fast Tac metabolism rate (C/D ratio) might be a risk factor for BKV infection. Recently, we showed that a fast Tac metabolism with a C/D ratio <1.05 ng/mL*1/mg was associated with a decreased renal function 24 months after RTx^12^. Study data suggested that a low C/D ratio is an independent risk factor for development of CNI nephrotoxicity or BKN. However, number of patients with BKN in that study was low. Therefore, we addressed this question in a larger cohort.

In the BKV group, most patients were identified as fast Tac metabolizers with a median Tac C/D ratio of 0.85 ng/mL*1/mg at 1 month after RTx. Logistic regression estimates verified that the C/D ratio 1 month after RTx is associated with BKV infection. Patients with histologically proven BKN were also identified to be fast Tac metabolizers with a median C/D ratio of 0.78 ng/mL*1/mg. Fast metabolizers might be exposed to over immunosuppression, toxicity or stimulation of BKV replication due to elevated Tac peak levels early after drug intake. In line with these observations, Barraclough *et al*. showed that median dose-adjusted exposure to tacrolimus was significantly higher in individuals carrying the NR1I2 8055T variant allele which encodes for a nuclear receptor that is involved in the detoxification and clearance of drugs, e.g. Tac, by acceleration of CYP3A metabolism. Patients carrying this allele are fast metabolizers with significantly higher odds of BK viremia^17^.

Patients in the BKV group were on average 3 years older compared to the control group and advanced age has been found to be a risk factor for BKV infection in former studies^2^,^11^,^18^,^19^. This is of note because younger age is associated with faster Tac metabolism^20^. Other risk factors for BKV infection like male gender^2^,^19^, Caucasian race^2^,^21^ and diabetes mellitus^21^ which have been reported could not be confirmed in our study.

A BK viral load >185,000 copies/mL at time of diagnosis was recently shown to be predictive for BKN^22^. In our study, patients with BKN had an initial median BK viral load of 37,627 copies/mL and a median maximum viral load of 441,700 copies/mL. BKV copies in BKN patients were markedly higher compared to the BKV patients without BKN (median initial viral load 4,334 and maximum viral load 18,572 copies/mL) suggesting that BKN is preceded by high-level BK viremia.

We herein found CMV status to be a risk factor for BKV infection. However, only 16.3% of patients in the BKV group experienced CMV infection. Of these, 50% of patients had CMV infection before BK viremia. Especially, the number of CMV high risk patients (D+/R−) was considerably higher in the BKV group. There are contradictory data on the association between CMV and BKV infection. Schachtner *et al*. reported CMV reactivation to be a risk factor for BKV replication^23^. In contrast, Elfadawy *et al*. suggested an indirect protective effect of CMV viremia against subsequent BK viremia^24^. In their prospective study CMV viremia was associated with a decreased incidence of BKV reactivation after kidney and kidney-pancreas transplantation, a finding which might be explained by the reduction of immunosuppression.

Due to rapid reduction of immunosuppressive therapy in BKV positive patients, renal function was not noticeably affected in the BKV group and in patients with histologically proven BKN after a 12 months follow-up. These data are in line with a prospective study by Schaub *et al*.^25^, in which renal function of patients with BK viremia, presumptive or definitive BKN was analyzed. Only patients with definitive BKN showed a temporary rise in creatinine after a median follow-up of 4 months after RTx and in all of them renal function completely recovered after 34 months^25^. However, in an OPTN analysis of national registry data, BKV treatment report was associated with a higher risk of subsequent graft loss^2^.

This study has a few limitations. Data were collected retrospectively and our study population exclusively includes patients of Western European descent. Thus, due to distinct genetic characteristics data might not be representative for other ethnical groups. Except for 3 protocol biopsies in 3 ABOi RTx recipients, renal biopsies were only performed on indication. Therefore, some cases of BKN might have been missed. This study was designed as a case-control study and patients with BKV infection during the first four years after renal transplantation were only included if data and follow-up were complete. Controls were randomly selected from patients that underwent RTx during the same time period and had a follow-up of at least four years. A possible selection bias could not be ruled out and prospective, multicentre studies are needed to confirm our results.

We herein showed that fast Tac metabolism is associated with BK viremia. Calculation of the Tac C/D ratio one month after RTx could assist to identify patients at higher risk for BK viremia early after RTx. Our findings may assist transplant clinicians in the decision process to individually tailor immunosuppression, especially if risk factors for BKV infection are present.

## Additional Information

**How to cite this article**: Thölking, G. *et al*. Influence of tacrolimus metabolism rate on BKV infection after kidney transplantation. *Sci. Rep.* **6**, 32273; doi: 10.1038/srep32273 (2016).

## Figures and Tables

**Table 1:** Patient’s characteristics.

	Control (n = 86)	BKV (n = 86)	P value
Age (years)	50.4 ± 14.6	53.4 ± 13.2	0.172^a^
Gender (male/female)	57 (66.3%)/29 (33.7%)	55 (64.0%)/31 (36.0%)	0.873^b^
Height (m)	1.72 ± 0.10	1.74 ± 0.09	0.248^a^
Weight (kg)	75.8 ± 13.8	74.7 ± 13.0	0.607^a^
BMI (kg/m^2^)	25.6 ± 4.4	24.6 ± 3.4	0.125^a^
Number of transplantation			0.949^b^
one	67 (77.9%)	69 (80.2%)	
two	15 (17.4%)	15 (17.4%)	
three	3	2	
four	1	−	
Living donor transplantation	19 (22.1%)	11 (12.8%)	0.159^b^
ESP	17 (19.8%)	23 (26.7%)	0.367^b^
ABOi	1	1	−
CIT (h)	9.2 ± 5.1	10.0 ± 5.4	0.315^a^
donor data			
donor age (years)	54.1 ± 13.0	53.8 ± 16.5	0.882^a^
donor gender (male/female)	38 (44.2%)/48 (55.8%)	39 (45.3%)/47 (54.7%)	1.0^b^
PRA (>20%)	5 (5.8%)	3 (3.5%)	0.720^b^
HLA MM			0.862^b^
no HLA MM	2	1	
1–3 HLA MM	36 (41.9%)	38 (51.4%)	
4–6 HLA MM	48 (55.8%)	46 (48.9%)	
CMV risk status			0.001^b^
CMV high risk	8 (9.3%)	26 (30.2%)	
CMV intermediate risk	73 (84.9%)	52 (60.5%)	
CMV low risk	5 (5.8%)	8 (9.3%)	
CMV infection	14 (16.3%)	14 (16.3%)	1.0^b^
CMV disease	10 (11.6%)	10 (11.6%)	1.0^b^
CMV before BKV infection	−	7/14 (50.0%)	−

Variables are reported as absolute and relative frequencies or mean ± standard deviation.

^a^t test for independent groups.

^b^Fisher’s exact test; Abbreviations: BMI, body mass index; ESP, European Senior Program; ABOi, ABO incompatible transplantation; CIT, cold ischemia time; PRA, panel reactive antibodies; HLA MM, human leucocyte antigen mismatch; CMV, cytomegalovirus; RTx, renal transplantation.

**Table 2:** Risk factors for BKV infection (multivariable analysis).

	Odds Ratio	95% Wald Confidence Limits (Wald test)		P value
Age (x vs. x-1 years)	1.03	0.99	1.05	0.067
log Tac C/D ratio 1 month (x vs. x-1 units)	0.37	0.20	0.71	0.003
CMV risk				0.002
high vs. low risk	2.43	0.48	12.43	0.285
intermediate vs. low risk	0.40	0.10	1.64	0.205
high vs. intermediate risk	6.03	2.17	16.78	0.001

Results of the logistic regression of potential risk factors for BKV infection within 4 years after RTx. Tac C/D ratio 1 month after RTx was log-transformed (natural logarithmic) to achieve equal intervals between C/D ratio units. Due to missing covariate values, 145 patients (71 BKV/74 controls) were entered in the analysis. Abbreviation: CMV, cytomegalovirus.

**Table 3:** Drug doses and blood levels.

	Control	BKV	P value
Tac mean trough level (ng/ml)			
after 1 month (n = 75/71)	11.1 ± 3.8	9.9 ± 3.6	0.017^a^
after 3 months (n = 70/67)	9.6 ± 3.0	8.7 ± 2.6	0.149^a^
after 6 months (n = 67/62)	8.3 ± 2.7	7.2 ± 2.1	0.012^a^
at BKV pos (n = 66)	−	8.9 ± 3.4	−
Tac mean daily dose (mg)			
after 1 month (n = 74/71)	9.0 (3–22)	11.0 (2–20)	0.064^b^
after 3 months (n = 69/67)	6 (1.5–22)	7 (2–20)	0.135^b^
after 6 months (n = 66/62)	4.5 (0.5–21)	5 (2–20)	0.082^b^
at BKV pos (n = 42)	−	7 (2–16)	−
Tac C/D ratio			
after 1 month (n = 74/71)	1.15 (0.28–5.05)	0.85 (0.30–8.75)	0.002^b^
after 3 months (n = 67/67)	1.58 (0.35–9.93)	1.16 (0.31–5.35)	0.017^b^
after 6 months (n = 66/62)	1.76 (0.43–7.20)	1.30 (0.44–4.20)	0.001^b^
at BKV pos (n = 37)	−	1.34 (0.33–3.95)	−
log Tac C/D ratio			
after 1 month (n = 74/71)	0.14 (−1.27–1.62)	−0.17 (−1.20–2.17)	0.002^b^
after 3 months (n = 69/67)	0.45 (−1.04–2.30)	0.15 (−1.18–1.68)	0.017^b^
after 6 months (n = 66/62)	0.56 (−0.85–1.97)	0.26 (−0.83–1.44)	0.001^b^
at BKV pos (n = 37)		0.29 (−1.11–1.37)	−
Tac metabolism groups			
after 1 month slow metab. (n = 69)	45 (61%)	24 (34%)	0.002^c^
fast metab. (n = 76)	29 (39%)	47 (66%)	
after 3 months slow metab. (n = 90)	51 (74%)	39 (58%)	0.070^c^
fast metab. (n = 46)	18 (26%)	28 (42%)	
after 6 months slow metab. (n = 93)	53 (80%)	40 (65%)	0.050^c^
fast metab. (n = 35)	13 (20%)	22 (35%)	
prednisolone daily dose (mg)			
after 1 month (n = 82/85)	20 (5–30)	20 (7.5–30)	0.089^b^
after 3 months (n = 81/85)	10 (5–20)	10 (5–50)	0.018^b^
after 6 months (n = 82/85)	5 (2.5–20)	5 (2.5–40)	0.058^b^
at BKV pos (n = 50)	−	10 (2.5–50)	−

For Tac dose and trough level calculation, only Tac twice daily was included. Results are reported as absolute and relative frequencies, mean ± standard deviation or median (minimum-maximum).

Abbreviations: BKV, BK virus; Tac, tacrolimus; C/D ratio, concentration/dose ratio; IS, immunosuppression.

^a^t test for independent groups.

^b^Mann Whitney U test.

^c^Fisher’s exact test; fast metabolism was defined as Tac C/D ratio <1.05, slow mebabolism was defined as Tac C/D ratio ≥1.05.

**Table 4:** Data on BKV and BKN.

BKV group (n = 86)	
BK viremia >7.000	46 (53.5%)
time from RTx until BKV pos (months)	6 (0–36)
initial BK viral load of BKV patients without BKN	4,334 (91–2,700,000)
maximum BK viral load of BKV patients without BKN	18,572 (91–3,100,000)
initial BK viral load of all BKV patients	5,540 (91–8,600,000)
maximum BK viral load of all BKV patients	25,446 (91–196,000,000)
patients on Tac prolonged release (Advagraf)	7
patients on MMF at BKV	44
MMF dose at initial BKV infection	1000 (500-2000)
Data of BKV patients with BKN (n = 8)	
Tac mean trough level (ng/ml) at time of BKN (n = 7)	7.8 ± 2.6
Tac mean daily dose (mg) at time of BKN (n = 7)	9.0 (3.0–15.0)
Tac C/D ratio (ng/mL*1/mg) at time of BKN (n = 7)	0.78 (0.33–2.47)
log Tac C/D ratio (log(ng/mL*1/mg)) at time of BKN (n = 7)	−0.24 (−1.12–0.90)
prednisolone mean daily dose (mg) at time of BKN (n = 6)	10 (5–20)
initial BK viral load (n = 7)	37,627 (850–8,600,000)
maximum BK viral load (n = 7)	441,700 (41,144–196,000,000)

Results are reported as absolute and relative frequencies, mean ± standard deviation or median (minimum-maximum). Abbreviations: BKV, BK virus; BKN, BK virus-associated nephropathy; viral load stated in copies/mL; Tac, tacrolimus; MMF, mycopheonolate mofetil.

**Table 5:** Adverse events and switch of immunosuppression.

	Control (n = 86)	BKV (n = 86)
Loss of function	4	4
Acute rejection	1	3
HUS recurrence	1	−
BKN and acute rejection	−	1
Chronic transplantat nephropathy	2	−
Switch from Tac to other IS	22/79	37/75
Everolimus	7	13
Tacrolimus extended release (Advagraf)	3	1
Cyclosporin A	6	20
Sirolimus	6	2
Tac termination without replacement	−	1
Cause of switch from Tac		
BKV infection	−	20
Malignoma	4	1
Alopecia	−	−
Tac blood level fluctuations	1	1
Bacterial infection	2	−
CMV infection	−	1
CNI nephrotoxicity	6	6
Diabetes mellitus	6	5
Incompliance	1	−
Neurotoxicity	1	3
HUS	1	−

Frequencies of adverse events and switch from Tac during the 4 year follow-up. Abbreviations: BKV, BK virus; Tac, tacrolimus twice daily; IS, immunosuppression; HUS, hemolytic uremic syndrome; BKN, BKV-associated nephropathy.

## References

[b1] Ekberg H. . Reduced exposure to calcineurin inhibitors in renal transplantation. N Engl J Med. 357, 2562–2575 (2007). 18094377 10.1056/NEJMoa067411

[b2] Dharnidharka V. R., Cherikh W. S. & Abbott K. C. An OPTN analysis of national registry data on treatment of BK virus allograft nephropathy in the United States. Transplantation 87, 1019–1026 (2009). 19352121 10.1097/TP.0b013e31819cc383

[b3] Hirsch H. H. . Prospective study of polyomavirus type BK replication and nephropathy in renal-transplant recipients. N Engl J Med. 347, 488–496 (2002). 12181403 10.1056/NEJMoa020439

[b4] Brennan D. C. . Incidence of BK with tacrolimus versus cyclosporine and impact of preemptive immunosuppression reduction. Am J Transplant 5, 582–594 (2005). 15707414 10.1111/j.1600-6143.2005.00742.x

[b5] Bressollette-Bodin C. . A prospective longitudinal study of BK virus infection in 104 renal transplant recipients. Am J Transplant 5, 1926–1933 (2005). 15996241 10.1111/j.1600-6143.2005.00934.x

[b6] Hirsch H. H. . Polyomavirus-associated nephropathy in renal transplantation: interdisciplinary analyses and recommendations. Transplantation 79, 1277–1286 (2005). 15912088 10.1097/01.tp.0000156165.83160.09

[b7] Hirsch H. H. BK virus: opportunity makes a pathogen. Clin Infect Dis. 41, 354–360 (2005). 16007533 10.1086/431488

[b8] Perez-Torres D. . Factors and outcome in BK virus nephropathy in a Hispanic kidney transplant population.Transpl Infect Dis. 12, 16–22 (2010). 19804584 10.1111/j.1399-3062.2009.00458.x

[b9] Suwelack B., Malyar V., Koch M., Sester M. & Sommerer C. The influence of immunosuppressive agents on BK virus risk following kidney transplantation, and implications for choice of regimen. Transplant Rev (Orlando) 26, 201–211 (2012). 21940156 10.1016/j.trre.2011.05.002

[b10] Dadhania D. . Epidemiology of BK virus in renal allograft recipients: independent risk factors for BK virus replication. Transplantation 86, 521–528 (2008). 18724220 10.1097/TP.0b013e31817c6447 PMC3647687

[b11] Hirsch H. H. . Polyomavirus BK replication in de novo kidney transplant patients receiving tacrolimus or cyclosporine: a prospective, randomized, multicenter study. Am J Transplant 13, 136–145 (2013). 23137180 10.1111/j.1600-6143.2012.04320.x PMC3563214

[b12] Tholking G. . The tacrolimus metabolism rate influences renal function after kidney transplantation. Plos One 9, e111128 (2014). 25340655 10.1371/journal.pone.0111128 PMC4207775

[b13] Tholking G. . Tacrolimus Concentration/Dose Ratio is Associated with Renal Function After Liver Transplantation. Ann Transplant 21, 167–179 (2016). 27003330 10.12659/aot.895898

[b14] Anglicheau D. . Pharmacokinetic interaction between corticosteroids and tacrolimus after renal transplantation. Nephrol Dial Transplant 18, 2409–2414 (2003). 14551375 10.1093/ndt/gfg381

[b15] Montero N. . Steroid avoidance or withdrawal for pancreas and pancreas with kidney transplant recipients. Cochrane Database Syst Rev. 9, CD007669 (2014). 10.1002/14651858.CD007669.pub2 PMC11129845 25220222

[b16] Hirsch H. H., Yakhontova K., Lu M. & Manzetti J. BK Polyomavirus Replication in Renal Tubular Epithelial Cells Is Inhibited by Sirolimus, but Activated by Tacrolimus Through a Pathway Involving FKBP-12. Am J Transplant 16, 821–832 (2016). 26639422 10.1111/ajt.13541 PMC5064607

[b17] Barraclough K. A. . NR1I2 polymorphisms are related to tacrolimus dose-adjusted exposure and BK viremia in adult kidney transplantation. Transplantation 94, 1025–1032 (2012). 23095803 10.1097/TP.0b013e31826c3985

[b18] Ramos E. . Clinical course of polyoma virus nephropathy in 67 renal transplant patients. J Am Soc Nephrol. 13, 2145–2151 (2002). 12138148 10.1097/01.asn.0000023435.07320.81

[b19] Mengel M. . Incidence of polyomavirus-nephropathy in renal allografts: influence of modern immunosuppressive drugs. Nephrol Dial Transplant 18, 1190–1196 (2003). 12748354 10.1093/ndt/gfg072

[b20] Stratta P. . The interactions of age, sex, body mass index, genetics, and steroid weight-based doses on tacrolimus dosing requirement after adult kidney transplantation. Eur J Clin Pharmacol. 68, 671–680 (2012). 22101623 10.1007/s00228-011-1150-0

[b21] Merlino C. . Polyomavirus BK DNA quantification assay to evaluate viral load in renal transplant recipients. J Clin Virol. 28, 265–274 (2003). 14522065 10.1016/s1386-6532(03)00012-x

[b22] Elfadawy N. . The impact of surveillance and rapid reduction in immunosuppression to control BK virus-related graft injury in kidney transplantation. Transpl Int. 26, 822–832 (2013). 23763289 10.1111/tri.12134

[b23] Schachtner T., Babel N. & Reinke P. Different risk factor profiles distinguish early-onset from late-onset BKV-replication. Transpl Int. 28, 1081–1091 (2015). 25959355 10.1111/tri.12601

[b24] Elfadawy N. . CMV Viremia is associated with a decreased incidence of BKV reactivation after kidney and kidney-pancreas transplantation. Transplantation 96, 1097–1103 (2013). 24056621 10.1097/TP.0b013e3182a6890d

[b25] Schaub S. . Reducing immunosuppression preserves allograft function in presumptive and definitive polyomavirus-associated nephropathy.Am J Transplant 10, 2615–2623 (2010). 21114642 10.1111/j.1600-6143.2010.03310.x

